# The expectations and experiences of patients regarding the diagnostic workup at a specialized memory clinic: An interview study

**DOI:** 10.1111/hex.14021

**Published:** 2024-03-21

**Authors:** Malin Aspö, Maria Sundell, Myroslava Protsiv, Fleur Wiggenraad, Marie Rydén, Francesca Mangialasche, Miia Kivipelto, Leonie N. C. Visser

**Affiliations:** ^1^ Division of Clinical Geriatrics, Department of Neurobiology, Care Sciences and Society, Center for Alzheimer Research Karolinska Institutet Stockholm Sweden; ^2^ Theme Inflammation and Aging, Medical Unit Aging Karolinska University Hospital Stockholm Sweden; ^3^ The Ageing Epidemiology Research Unit, School of Public Health Imperial College London London UK; ^4^ Institute of Public Health and Clinical Nutrition University of Eastern Finland Kuopio Finland; ^5^ Department of Medical Psychology, Amsterdam UMC University of Amsterdam Amsterdam The Netherlands; ^6^ Amsterdam Public Health Research Institute Quality of Care Amsterdam The Netherlands; ^7^ Alzheimer Center Amsterdam, Neurology, Vrije Universiteit Amsterdam, Amsterdam University Medical Center VU University Medical Center Amsterdam The Netherlands; ^8^ Amsterdam Neuroscience Neurodegeneration Amsterdam The Netherlands

**Keywords:** Alzheimer's disease, dementia, diagnostic work‐up, memory clinic, physician–patient communication, subjective cognitive decline

## Abstract

**Background:**

Because of the shift towards earlier diagnosis of dementia and/or Alzheimer's disease (AD), increasing numbers of individuals with subjective cognitive decline (SCD) and mild cognitive impairment (MCI) are seen in memory clinics. Yet, evidence indicates that there is room for improvement when it comes to tailoring of the diagnostic work‐up to the needs of individual patients. To optimize the quality of care, we explored patients' perspectives regarding the diagnostic work‐up at a specialized memory clinic.

**Methods:**

This interview study was conducted at Karolinska University Hospital (Sweden). The comprehensive diagnostic work‐up for dementia at the memory clinic in Solna is conducted within 1 week. A sample of 15 patients (8 female; mean age = 61 years [range 50–72]; 11 SCD, 1 MCI and 3 AD dementia) was purposively selected for a series of three semistructured interviews, focussing on (1) needs and expectations (during the week of diagnostic testing), (2) experiences (within 2 weeks after test‐result disclosure) and (3) reflections and evaluation (3 months after disclosure). Transcribed audio‐recorded data were analyzed using thematic content analysis (using MaxQDA software).

**Results:**

Three key themes were identified: (1) the expectations and motivations of individuals for visiting the memory clinic strongly impacted their experience; (2) the diagnostic work‐up impacted individuals psychosocially and (3) the diagnostic work‐up provided an opportunity to motivate individuals to adopt a healthier lifestyle.

**Conclusion:**

Our findings underscore the importance of enquiring about the expectations and needs of individuals referred to a specialized memory clinic, allowing for expectation management and personalization of provided information/advice, and potentially informing the selection of patients in need of a comprehensive diagnostic work‐up. Structural guidance might be needed to support those with SCD and MCI to help them cope with uncertainty, potentially resolve their issues, and/or stimulate brain health.

**Patient or Public Contribution:**

We gathered the perspectives of 15 individuals who had been referred to the memory clinic at three different time points through semistructured interviews, and these interviews were the primary data source.

## 1 INTRODUCTION

Dementia is a leading cause of mortality and one of the most important causes of dependency and disability amongst older people worldwide.^1^ Dementia can be caused by a number of diseases that damage the brain, with Alzheimer's disease (AD) as the most common one.^2^ Over the last few decades, we have gained a deeper understanding of the pathological processes in the brain that occur with AD, which can start up to 25 years before the onset of dementia.^2^ This insight and the advances made in diagnostic testing resulted in a shift of focus towards earlier disease stages,^3^ since these provide a window of opportunity to intervene by means of multidomain lifestyle interventions or disease‐modifying treatment aimed at slowing down disease progression.^4^, ^5^

This shifting focus to earlier disease stages and the continuing progress in the development of diagnostic and genetic testing results in increasing numbers of people with memory problems or other cognitive complaints being referred to a memory clinic for a diagnostic work‐up of dementia. However, cognitive decline can be caused by a variety of diseases and conditions, meaning that only a minority of patients will meet the criteria for dementia. The proportion of individuals with mild cognitive impairment (MCI), that is, those with objective cognitive deficits without significant impact on activities of daily living, and subjective cognitive decline (SCD), that is, those with worries about their cognitive functioning, yet without objective cognitive impairment, is substantial.^6^ This is especially true in specialized memory clinics with a comprehensive diagnostic workup, that often cater to a younger population,^6^ such as the memory clinic at Karolinska University Hospital in Solna (Sweden) where newly‐referred patients are on average 59 years old and approximately half of them are diagnosed with SCD and a quarter with MCI.^7^ In those individuals, AD biomarker and genetic testing could inform a prognosis, that is, the risk of developing dementia due to AD.^4^, ^8^ However, these test results do not provide certainty and are difficult to interpret, communicate and comprehend.^8^ The information provided throughout the diagnostic trajectory and as a result of such a diagnostic work‐up could therefore have both positive, as well as negative consequences.^9^

How individuals are impacted and how they experience the diagnostic trajectory, including the communication about test results, might depend on their expectations and motivations for visiting the memory clinic.^9^, ^10^ Only a small proportion of individuals visiting a memory clinic want to confirm or exclude a dementia diagnosis.^11^ Most individuals want to find out what is causing their symptoms or subjective complaints, irrespective of their syndrome diagnosis, and many of them have specific motivations for visiting the memory clinic.^11^ They, for example, seek (more) information about their current diagnosis or prognosis, access to specific care service(s) and treatment and/or advice on how to cope with their current symptoms.^11^ Ideally, the diagnostic workup and provided information should therefore be tailored to the individual patient's needs, expectations and situation.^12^

Some evidence indicates that there is room for improvement when it comes to tailoring of the diagnostic work‐up, including communication with patients.^13^, ^14^ A Dutch, multicentre study, has observed that patients' and their care partners' motivations for visiting the memory clinic were not addressed in more than half of their first consultations at the memory clinic.^11^ In addition, many aspects that were deemed important to discuss throughout the diagnostic trajectory,^15^ were found not to be addressed,^16^ for example, information about what people can expect in terms of test outcomes, information about prognosis and the implications of certain test results. This explains why patients and care partners frequently report unmet information needs.^17^ Evidence also shows that patients and care partners rarely ask questions, express their preferences, or initiate discussion of a topic in consultation with their doctor,^11^, ^18^ which leaves us wondering about their expectations and experiences throughout the diagnostic trajectory.

Most of the research so far that has been conducted in the memory clinic setting has focused on observations from a specific clinical consultation, or quantitative data collected by means of questionnaires often only assessing short‐term psychological impact of the disclosure of certain test results.^9^, ^19^ Currently, *in‐depth* insight into patients' expectations and experiences throughout the diagnostic work‐up for dementia at the memory clinic is lacking. Such insight could inform practice recommendations on how to organize memory clinic care, including how to communicate with patients during the diagnostic trajectory, to ultimately improve quality of care and patient outcomes and experiences. This might be especially relevant for the more specialized memory clinics that cater towards a large majority of patients who do not (yet) meet the criteria for dementia. This interview study therefore aims to explore patients' expectations and experiences throughout the diagnostic work‐up for dementia at a specialized memory clinic.

## 2 METHODS

### 2.1 Design and study context

This interview study was part of a larger research project entitled the ‘Medical Communication at Memory Clinics' (MedKom) project. This project was conducted at the memory clinic(s) connected to the Karolinska University Hospital in the Stockholm area of Sweden, in the context of the projects EU‐Fingers (www.eufingers.com) and LETHE (www.lethe-project.eu). The main objective of the MedKom project is to assess the impact of and identify best‐practices for, communicating about AD biomarkers and dementia risk in memory clinics. To get an in‐depth understanding of patients' perspectives regarding the diagnostic trajectory, interviews were conducted in a subsample of the MedKom participants. The study was reviewed and approved by the Ethics Review Board in Stockholm, Sweden (dnr: 2021‐02882). In accordance with the declaration of Helsinki,^20^ participants were informed that participation was voluntary, and no interviews were conducted before obtaining written consent from all participants.

### 2.2 Setting

The memory clinic at Karolinska University Hospital in Solna is a specialized memory clinic, primarily seeing patients with cognitive complaints/symptoms that are younger than 70 years of age. The clinic opened in April 2018 and applies a fast‐track model, in which all assessments are performed within 1 week by a team consisting of physicians, neuropsychologists, nurses, a physiotherapist and a speech‐language pathologist. Patients are referred to the clinic after they have undergone a basic evaluation (e.g., including a Mini‐Mental State Examination [MMSE], a general physical evaluation, blood test and sometimes a brain‐computer tomography scan), usually by their general practitioner. The standardized, fast‐track diagnostic work‐up at the memory clinic includes a medical and neurological examination including (hetero‐) anamnesis, lab blood tests, neuropsychological assessment and AD biomarker testing, using magnetic resonance imaging and cerebrospinal fluid, following the national guidelines from the Swedish Board of Health and Welfare. On average, about three in four patients referred to this clinic are diagnosed with SCD or MCI, and one in four with dementia.^7^

### 2.3 Participants

Participants for the MedKom project were recruited consecutively between October 2021 and June 2022. To be eligible, the newly‐referred patient needed to have sufficient knowledge of the Swedish or English language and not to have severe cognitive impairment upon referral (i.e., those with MMSE scores < 18 and those not able to provide informed consent were excluded). Also, patients who were seen at the clinic for a second opinion (based on test results that were already available) or for neuropsychological assessment only in the context of Parkinson's disease, were excluded because they did not follow the standardized procedure. In the MedKom project, 78 out of 106 (74%) eligible patients were included at the Solna memory clinic (59 ± 5 years, 50% female), who received the following diagnoses: 51 SCD with negative AD biomarkers, 10 MCI with negative AD biomarkers, 3 MCI with positive AD biomarkers, 11 dementia and 3 other. A subsample of these participants was asked (face‐to‐face) to take part in the current interview study (including three interviews). These participants were purposefully selected to generate a heterogeneous sample based on sex and age. Participants were recruited until data saturation was achieved, for example, no new information was mentioned in subsequent interviews.

### 2.4 Procedures: Interviews and other measures

Participants were interviewed at three different time points, to investigate expectations and experiences thoroughly and without the bias that would occur if we would only ask people to recall their expectations and experiences retrospectively at one point in time. We used a semistructured approach and an interview guide (see Supporting Information S1: Appendix A). The focus of the first interview was on the person's needs and expectations (example question: ‘What is most important to you to get out of the diagnostic work‐up?’). This interview took place on one of the days between the participant's first visit to the clinic (on Mondays) and the test result disclosure consultation (on Fridays). The second interview was conducted within 2 weeks after the test result disclosure consultation, and focused on the person's experiences at the clinic, for example, regarding perceived communication with the staff at the clinic and the impact of receiving information about test results and diagnosis (example questions: ‘What could the staff have done differently to better meet your needs?’ and ‘How has the (result of the) diagnostic workup impacted you/your life?’). After 3 months, a third interview was conducted to reflect on the diagnostic work‐up at the clinic and evaluate the long‐term impact of the diagnostic work‐up and the provided information regarding (biomarker) test results, diagnosis and dementia risk (example questions: ‘How do you now feel/what do you think about the doctor's communication’ and ‘How has the diagnostic work‐up impacted you/your life?’). The interviews were conducted by phone, by three different members of the study team (M. S., F. W., M. A.; two physicians and one research nurse, all female). The first and second interviews were most often conducted by the same team member, yet the third interview could be conducted by a different study team member. All interviews were audio recorded. One participant had his study partner (wife) attending the first and second interviews; however, for this study, we have only analyzed the patient's part of these interviews.

In addition to the interviews, participants were asked to report their age, sex and years of education in a survey administered at their first visit to the memory clinic. Patients' MMSE and Montreal Cognitive Assessment (MoCA) scores were retrieved from their medical records (range 0–30, with higher scores indicating better cognitive function). Patients were retrospectively categorized into diagnostic categories based on data retrieved from their medical records: (1) dementia, (2) MCI or (3) SCD, with or without the presence of AD pathology.

### 2.5 Analyses

The audio‐recorded interviews were transcribed by a research assistant not involved in the data collection and M. S. checked the accuracy of all transcripts. MAXQDA 12 software^21^ was used to assist in the coding process and identification of themes. We conducted an inductive, thematic content analysis aimed at generating coding categories from the data.^22^ During the phase of familiarization M. S., M. P. and M. A. read all transcripts repeatedly, which were grouped per participant, searching for patterns and initial coding categories. The interviews were then coded independently by M. S., M. P. and M. A. (a physician, a researcher and a research nurse, all female). Each interview was coded by at least two of the coding members. The study team had regular meetings to compare categories and decide on categories and subcategories emerging from the data. In case of disagreement, the third person was involved to reach a consensus. During the process, the coders went back and forth between the data set and codes to refine the (sub)categories. Finally, in several consensus meetings and rounds of feedback on preliminary reports, the resulting categories were discussed and grouped, to synthesize the most relevant findings into overarching themes, eventually resulting in the key themes. The analysis process has been guided by L. N. C. V. (female senior researcher, with ample experience in qualitative research). All authors critically reviewed the key themes, resulting in minor changes and the final key themes as now reported in this manuscript.

## 3 RESULTS

### 3.1 Participant characteristics

Fifteen patients (eight female/seven male) were interviewed. The average age was 60.8 years (ranging from 50–72), education 14.6 years (range 11–19), and they had an average MoCA score of 24.7 (range 20–29) and MMSE score of 27.3 (range 23–30). The most common syndrome ‘diagnosis’ was SCD (11/15, 73%), one participant received an MCI diagnosis, and three participants were diagnosed with dementia due to AD. Only the participants with dementia had positive AD biomarkers.

Out of 15 participants, 13 completed all three interviews. Overall, only two interviews were missing: for one participant (ID = 014) the first interview was missing, and for another participant (ID = 013), the second interview, both due to scheduling difficulties within the set time frame. So, 43 interviews were conducted in total. Interviews lasted on average 24 min (ranging from 11 to 44 min).

### 3.2 Themes

We identified three key themes:

1. The expectations and motivations of individuals for visiting the memory clinic strongly impacted their experience.
2. The diagnostic work‐up impacted individuals on psychological and social levels.
3. The diagnostic work‐up at the memory clinic provided an opportunity to motivate individuals to adopt a healthier lifestyle.

#### 3.2.1 The expectations and motivations of individuals for visiting the memory clinic strongly impacted their experience

First, independent of the diagnosis they received and the level of reported satisfaction with the diagnostic work‐up itself, participants expressed that they felt included in the diagnostic process and that their concerns were taken seriously. However, participants could neither recall if anyone at the clinic had explicitly asked them about their personal expectations or preferences regarding diagnostic testing nor expected the clinic staff to do so.

Yes but, it's like, if you have agreed to undergo the investigation and all that, then you should take all the tests that are part of it. In that way I've been involved, in that I've said, yes sure. I don't know. How could one be more involved? (ID_009 interview 1, MCI)

The experience of the diagnostic trajectory mainly depended on participants' motivations for seeking care and their expectations of getting the answers they were looking for. Common motivations for seeking care were that they wanted clarity regarding (the cause of) symptoms, information and advice on how to manage symptoms, and/or treatment. Participants who expected treatment and the ‘problem to be fixed’ were less confident from the start that the diagnostic work‐up would provide them with the answers they were looking for, compared to participants whose motive was to rule out or confirm dementia. If not possible to treat, participants hoped that the diagnostic workup would at least provide them with prognostic information (if and how symptoms would progress) and tools for handling symptoms in daily life.

Part of it is finding out what it's about, what's wrong, so you can do something about it, and kind of find your way back, or get it back, that memory and that cognition that you've had in the past as well. […] But those are kind of high expectations […]. (ID_012 interview 1, SCD)

The level of satisfaction with the provided care was strongly related to participants' motivations and expectations. Participants having an expectation that was perfectly in line with what the clinic could offer, for example, confirming or ruling out dementia, were in general satisfied with the medical information provided, independent of the diagnosis or diagnostic label they received.

Apart from the outcome [being diagnosed with AD dementia], my experience was great. Eeh, it's been like that throughout the entire process, I felt welcome and, yes, the way I was informed about the diagnosis, and things like that, I think has been great. (ID_015 interview 2, AD dementia)

However, participants' experiences did not always match their expectations. This was more pronounced amongst participants who ended up receiving a ‘diagnostic label’ of SCD. Their motivation for seeking care was often related to an expectation of getting a clear answer about what is causing their complaints and receiving a plan to move forward, not only to rule out dementia. Not receiving a causal explanation for their cognitive complaints in the end, nor a treatment or (structural) guidance on how to manage symptoms, was experienced as frustrating. Participants also expressed disappointment over being referred back to their general practitioner, as they had hoped that the experts at the specialized memory clinic would have provided a solution for their issues/problems.

That there were sort of ideas of what you could do, how you could continue. Because what they're doing now, it's that they refer back to the eeh, well, regular doctors and those are not experts in this field […]. And then I'm thinking that maybe there would be some sort of expert way forward on this theme. And not having to go back to square one basically. Since the general practitioner is not an expert on this. (ID_ 012 interview 3, SCD)

#### 3.2.2 The diagnostic work‐up impacted individuals on psychological and social levels

Participants shared their thoughts about how the diagnostic work‐up had impacted their life both short and long‐term. Most participants whereby dementia was ruled out expressed a sense of relief and reported decreased levels of anxiety both in shorter and longer term.

I've put it behind me actually quite a bit. I was worried before, but I don't think much about it anymore. (ID_003 interview 3, SCD)

They also described a change in self‐esteem and confidence in the sense that knowing that they do not have dementia made them more accepting when they experienced cognitive problems, for example, when forgetting something. Some described how the results of the diagnostic workup also impacted their social connections, for example, changing jobs or increasing their social endeavours (again), after ruling out dementia or AD as a cause for their cognitive complaints.

I can say that I have changed jobs, and so, these symptoms that my family had noticed, they are gone now. (ID_007 interview 3, SCD)

[after I received results of the diagnostic work‐up] I realize that I needed to sort of get a grip on social connections again […] I'm part of a sewing circle […] so then I decided to go to this sewing circle again, very reluctantly I might add. But still I went. And when I left there, I realized how we just had so much, so much fun. I mean we laughed until we cried. (ID_002 interview 2, SCD)

However, some of the participants who perceived not having received a clear explanation for their symptoms and no treatment or guidance on how to proceed still reported feeling anxious due to not knowing what was going to happen next and the perceived lack of structural follow‐up.

Was this it, well, what happens now? What, what should I do next, what should I do with this memory problem? How, yes, what should I do? Who is it that should answer about what is to come, or about the future, or what opportunities there are to either continue to look for causes or to do something about it. […] But, but now what? What should I do now? (ID_006 interview 2, SCD)

In those who received a diagnosis of dementia, a process of grieving and acceptance was observed, slowly coming to terms with the diagnosis and their future looking different than expected. Nevertheless, although participants experienced that they had worked through the immediate feelings of sadness and grief, the concerns about their uncertain long‐term perspective remained.

The three months that have passed at home, have been transformative. And it still is. It comes and goes. I also allow myself to be sad sometimes. Like, it's a disease that I'm dying from as well, and I don't know how many years I have left and I'm still only 54. I think it's unfair and all this stuff that you have to go through. I also know that this process is important to go through and not skip. So, I will let it take its time, but not too long. (ID_004 interview 3, AD Dementia)

Well, quite a lot has happened here in the last few weeks. […] More difficult at the beginning, now I have informed a lot of people who are, who are close to me and so on. Which means that, yes, suddenly it feels like it's all right. Eh, I am thinking about what I'm going to work on in the future and, and sort of changing my role at work, and everyone that I've talked to so far is very understanding and receptive and supportive and stuff. So that, it's rather the long‐term perspective which is the concern. […] It is probably the long‐term perspective that is uncertain, so to speak. (ID_015 interview 2, AD dementia)

Two of the three participants receiving a diagnosis of dementia due to AD talked about being more intentional with how, and with whom, they want to spend their time and energy.

I now take the opportunity to spend more time with family […] Instead of sitting there reading the newspaper. I can just as well do that when they're not at home. […] I would say that I do fewer things alone now. (ID_015 interview 2, AD dementia)

Spirituality was explored as a resource for coping with these difficult times in life. Spirituality was defined broadly by participants, for example, using words like higher power, another dimension, creator energy, peace of mind, what makes us human/soul, positive outlook on life, quiet reflection, respect, feeling good while exercising/being in nature, inner stillness, finding meaning, meditation, relaxation/calmness, higher being and transcendence. There was an overall positive view of spirituality and a belief that it can be beneficial. However, not everyone reported to have the ability to draw from spirituality for coping with their current symptoms/issues. One participant for example identified as spiritual but described a lack of access to this spirituality for coping with hard times, and another participant currently identified as non‐spiritual yet expressed a desire to become more spiritual to be able to use it in daily life.

I don't think I am, I'm not a spiritual person so to speak. So, I would like to be more spiritual, that of course. I know it would have a positive effect on me if I would be, uhm, if I would practice some spirituality. (ID_009 interview 3, MCI)

#### 3.2.3 The diagnostic work‐up at the memory clinic provided an opportunity to motivate individuals to adopt a healthier lifestyle

Most participants mentioned that healthy lifestyle choices were addressed in the consultation(s) with their physician at the memory clinic and some clearly denoted having received individualized advice. Areas that were most often mentioned were exercise, diet, stress management, sleep and alcohol intake. Some participants felt that the communication with their doctor and the diagnostic work‐up in itself were a clear trigger for them to change their lifestyle. Some expressed that they had already been reflecting on their lifestyle quite a lot by themselves before the work‐up, and how they now got a little boost to actually make improvement to their lifestyle or maintain their efforts.

Speaking of health effects, thinking of what one eats and drinks and that kind of stuff, that is affected of course, and I think more often about that now […] my thoughts go back to those discussions I had with you [the clinic staff]. And now it's time to actually do it too. (ID_005 interview 3, SCD)

Most of the participants who expressed motivation to change their lifestyle after the disclosure consultation confirmed having made some changes by the 3‐month interview. The most common change was increasing exercise, to varying degrees.

I have started exercising a bit, something I have never done before. (ID_010 interview 3, SCD)

It's like, I feel that, that if there is something I can do to sort of, ehm, help keep a diagnosis at bay for a while then I am more motivated to do so now than before. Before it was more like ‘oh well, it will come when it comes’. (ID_011 interview 3, SCD)

One participant clearly stated a wish/need for concrete help to achieve lifestyle change and disappointment with not receiving such support.

I would have wanted maybe more concrete solutions on groups, contexts, support. Rather than just two brochures. […] I'm looking for a program […] So I can get help and support and cheering on. (ID_012 interview 2, SCD)

## 4 DISCUSSION AND CONCLUSION

### 4.1 Discussion

In this interview study, we gathered in‐depth insight on the perspectives of individuals who visited a specialized memory clinic and underwent a comprehensive diagnostic work‐up. Results showed that the expectations and motivations of patients for visiting the memory clinic shaped their experience and evaluation of the diagnostic trajectory. In addition, we found that the diagnostic work‐up impacted people psychosocially, in both positive and negative ways, not necessarily dependent on the outcome of the diagnostic work‐up in terms of syndrome diagnosis. One of the positive consequences was that many patients felt encouraged to improve their lifestyle to prevent (further) cognitive decline, based on the information and (tailored) advice given by their memory clinic physician. These findings highlight the importance of a personalized approach to memory clinic care and a need for multidomain (lifestyle) interventions aiming at reducing the risk of cognitive decline and dementia.^5^, ^23^

Previous research has focused on disclosing and receiving a dementia diagnosis^24^ and/or the safe disclosure of abnormal results of biomarker tests for AD in earlier disease stages, highlighting the importance of a process approach with pre‐diagnostic testing counselling and follow‐up.^25^ However, the number of people with concerns about their brain health and/or those MCI and SCD seems to increase and make up for a considerable proportion of individuals referred to the more specialized memory clinics.^6^, ^7^ Those patients do often not receive a clear diagnosis or aetiological explanation for their cognitive complaints and in our interviews, they reported feelings of disappointment and sometimes frustration with the perceived lack of specific information, personalized advice or treatment and concrete guidance or structural follow‐up. These findings are in line with a recent literature review, indicating that receiving normal results from biomarker testing for AD can evoke frustration and disappointment in addition to feelings of relief or reassurance.^9^, ^19^ Most patients come to the clinic in hopes of finding clarity about the cause of their complaints and a clear plan forward, regardless of the syndrome diagnosis.^10^, ^11^ However, the diagnostic work‐up does not always offer the certainty patients and their care partners were seeking, since uncertainty remains an inherent aspect of diagnostic testing and its outcome, especially in SCD and MCI.^17^, ^26^, ^27^ Clinicians also indicate to struggle with communicating uncertainty and specific support on how to communicate about, and cope with, uncertainty might thus be warranted for clinicians as well as patients and their relatives.^28^ However, the offering in terms of treatment, guidance, and/or counselling for those with SCD or MCI, without any evidence of a neurodegenerative disease such as AD being present, is currently limited. At the more specialized memory clinics, the extensive nature of the diagnostic workup might contribute to the expectation that the experts will find out what is wrong and be able to ‘fix’ the problem(s). It thus seems particularly important to clarify the aim of the diagnostic work‐up, the possible outcomes including potential uncertainty, and the corresponding healthcare offer early in the diagnostic trajectory to manage expectations, maybe even at the time of referral.^17^

A process of shared decision making regarding whether or not to enter the diagnostic process at a (specialized) memory clinic and about which diagnostic test to undergo, would enable a two‐way interaction between health care providers and patients, discussing what is most important to individuals and what their needs are, as well as what the clinic can and cannot offer.^12^, ^17^ Such a process would result in a shared agreement on how to proceed, what information to provide, and which outcomes to achieve. In addition to better‐informed decisions aligned with what is most important to patients,^29^ shared decision making could improve patient outcomes, for example, resulting in adequate levels of knowledge and satisfaction, and potentially even helping to reduce unnecessary diagnostic testing.^30^ Both general practitioners as well as memory clinic physicians could play an important role in facilitating the process of shared decision making,^31^, ^32^ yet Berger et al.^33^ also emphasize the importance of patient participation in these discussions, which could be encouraged through the use of decision aids.^32^

In addition to physician–patient consultations, written or digital information might assist in managing expectations before, and/or aiding personalized information provision and advice after the diagnostic work up.^34^ Clear information sheets about common reasons for (subjective) cognitive decline such as sleep disturbances and heightened stress levels could be helpful, especially if combined with advice on what to do to manage these alternative causes for an individual's cognitive complaints, or where to specifically seek further assistance.^35^ A written summary of the test results and syndrome diagnosis, paired with information on the probable cause, could also prove useful for patients when communicating with their general practitioner or loved ones and to serve as a source of information to come back to if questions arise.^36^

Regardless of the syndrome diagnosis and experience of the diagnostic trajectory, there seems to be a window of opportunity to motivate patients to adopt a healthier lifestyle. Considering that patients with SCD and MCI are at increased risk of developing dementia in the future,^37^, ^38^ such lifestyle change seems particularly important in terms of prevention of (further) cognitive decline. Although disease‐modifying treatment for AD might also be available in the near future, most of the patients currently seen at memory clinics do not meet the eligibility criteria for such anti‐amyloid treatment, even in a memory clinic setting with all necessary biomarker assessments in place.^7^ This was also reflected in our sample, with a large proportion of individuals with SCD and MCI, without AD pathology present. For those individuals, lifestyle change might help to reduce current symptoms and prevent cognitive decline, making it a valuable avenue for intervention.^39^, ^40^ Adequate knowledge about, and motivation for, making positive lifestyle changes might also give patients a sense of agency and control. The diagnostic workup at the memory clinic can thus be used to identify and motivate individuals for timely risk‐reducing interventions.^23^ An optimal approach would be to directly refer patients to such a program or other forms of concrete support to help initiate change and maintain a healthier lifestyle, which could also serve to reduce patients' feelings of being ‘left alone to deal with their problems' after a dementia diagnosis has been excluded.

### 4.2 Strengths and limitations

Amongst the strengths of this study is its qualitative design, allowing us to truly explore the perspectives of a heterogeneous sample of patients visiting the memory clinic. Moreover, we were able to gain rich insights, since we interviewed participants at three different time points, enabling us to collect data on their expectations, actual experiences, immediate impact, and reflections/evaluations after 3 months and compare these. In addition, the team interviewing and coding the data consisted of a diverse group of experienced researchers with backgrounds in behavioural and/or public health sciences, as well as those with a clinical background as a medical doctor or nurse, allowing us to reflect on the data and our own role in data analysis from various angles. We reported on our research according to the COnsolidated criteria for REporting Qualitative research‐criteria for qualitative research.^41^ Some limitations deserve mentioning. First, in this explorative, qualitative study, we included patients from one specialized clinic only, based in Sweden, and we, therefore, do not know how our findings translate to other clinics, populations, or countries. Second, we did not return the interview transcripts or the results to our participants for comments or feedback. Third, we cannot exclude potential selection bias, in the sense that people who did not want to participate (e.g., because they were overwhelmed by everything going on already) or those who could not participate (e.g., because they were not able to comfortably speak English or Swedish), could have reported different expectations and/or experiences.

### 4.3 Future research directions

The memory clinic that took part in the current study, provides all patients with detailed test results as part of their standard routine, explains potential factors contributing to the experienced cognitive symptoms, and provides advice on things that people could do/change to help solve their problems. Yet, some individuals still expressed frustration and dissatisfaction with the information and support that was offered. We need to find out how we could optimally communicate with those individuals about their subjective or mild cognitive decline, the probable/potential contributing factors, their dementia risk and the inherent uncertainty, in a way that resonates with them, so that they feel heard and better equipped to cope with the diagnostic and prognostic uncertainty. In addition, future research should further explore how to optimally design care services for those at increased risk of developing dementia. Such programs should be tailored to individuals' biomedical characteristics, yet also to their information needs and personal situation,^4^, ^5^ and should target multiple domains.^23^ Spirituality could, for example, be explored as a protective factor and coping source in a subpopulation of patients.^42^, ^43^ We also need to investigate whether memory clinics are the best setting to implement these services or if we need to consider other settings to provide such ‘brain health services’.^44^ Finally, we need to (further) develop and implement educational materials and communication tools in co‐creation with relevant stakeholders (such as www.ADappt.health), that could help manage patients' expectations, provide information adequately, and support patient participation during medical consultations and the process of shared decision making.^45^

## 5 CONCLUSION

The field of AD and dementia has shifted focus to earlier disease stages resulting in increasing numbers of people with SCD or MCI being referred to memory clinics for a diagnostic work‐up. Our findings underscore the importance of a timely conversation on personal needs and expectations, allowing for expectation management and personalization of provided information/advice, to align with the personal needs of these individuals. Our results also highlight that the memory clinic staff‐patient dialogues that are held during the diagnostic work‐up offer excellent opportunities to encourage individuals to adopt a healthier lifestyle to possibly prevent or delay (further) cognitive decline. Yet, structural guidance might be needed to support those with SCD and MCI and acknowledge their needs, help them cope with the inherent uncertainty, potentially resolve their issues, and/or stimulate their brain health.

## AUTHOR CONTRIBUTIONS

**Malin Aspö**: Investigation; writing—original draft; writing—review and editing; formal analysis; project administration; conceptualization. **Maria Sundell**: Investigation; writing— original draft; writing—review and editing; formal analysis; project administration; conceptualization. **Myroslava Protsiv**: Writing—original draft; writing—review and editing; formal analysis; conceptualization. **Fleur Wiggenraad**: Investigation; writing—review and editing. **Marie Ryden**: Writing—review and editing; validation; resources. **Francesca Mangialasche**: Writing—review and editing; validation; resources; funding acquisition; supervision. **Miia Kivipelto**: Funding acquisition; writing—review and editing; supervision; resources. **Leonie N. C. Visser**, Conceptualization; funding acquisition; writing—original draft; writing—review and editing; methodology; formal analysis; supervision; resources.

## CONFLICT OF INTEREST STATEMENT

Leonie N. C. Visser has been invited as a speaker for the Schwabe Group and her research has been funded by ZonMW, Health Holland, the Amsterdam Public Health Research Institute, Alzheimer Netherlands and private partners, including Eisai. All fees and funding are paid to her institution. The remaining authors declare no conflict of interest.

## ETHICS STATEMENT

The study was reviewed and approved by the Ethics Review Board in Stockholm, Sweden (dnr: 2021‐02882). In accordance with the declaration of Helsinki, participants were informed that participation was voluntary, and no data were collected before obtaining written consent from all participants.

## Supporting information

Supporting information.

## Data Availability

Research data are not shared (for privacy reasons).

## References

[1] World Health Organization . Global Status Report on the Public Health Response to Dementia. WHO; 2021.

[2] Scheltens P , De Strooper B , Kivipelto M , et al. Alzheimer's disease. Lancet. 2021;397:1577‐1590. 33667416 10.1016/S0140-6736(20)32205-4 PMC8354300

[3] Jack Jr. CR , Bennett DA , Blennow K , et al. NIA‐AA research framework: toward a biological definition of Alzheimer's disease. Alzheimers Dement. 2018;14:535‐562. 29653606 10.1016/j.jalz.2018.02.018 PMC5958625

[4] Frisoni GB , Altomare D , Ribaldi F , et al. Dementia prevention in memory clinics: recommendations from the European task force for Brain Health Services. Lancet Reg Health Eur. 2023;26:100576. 36895446 10.1016/j.lanepe.2022.100576 PMC9989648

[5] van der Flier WM , de Vugt ME , Smets EMA , Blom M , Teunissen CE . Towards a future where Alzheimer's disease pathology is stopped before the onset of dementia. Nat Aging. 2023;3:494‐505. 37202515 10.1038/s43587-023-00404-2

[6] van der Flier WM , Scheltens P . Amsterdam dementia cohort: performing research to optimize care. J Alzheimers Dis. 2018;62:1091‐1111. 29562540 10.3233/JAD-170850 PMC5870023

[7] Rosenberg A , Öhlund‐Wistbacka U , Hall A , et al. β‐Amyloid, tau, neurodegeneration classification and eligibility for anti‐amyloid treatment in a memory clinic population. Neurology. 2022;99:e2102‐e2113. 36130840 10.1212/WNL.0000000000201043 PMC9651451

[8] Visser LNC , Minguillon C , Sánchez‐Benavides G , et al. Dementia risk communication. A user manual for Brain Health Services—part 3 of 6. Alzheimers Res Ther. 2021;13:170. 34635169 10.1186/s13195-021-00840-5 PMC8507171

[9] van der Schaar J , Visser LNC , Ket JCF , et al. Impact of sharing Alzheimer's disease biomarkers with individuals without dementia: a systematic review and meta‐analysis of empirical data. Alzheimers Dement. 2023;19:5773‐5794. 37496313 10.1002/alz.13410

[10] Linden I , Hevink M , Wolfs C , Perry M , Dirksen C , Ponds R . Understanding patients' and significant others' preferences on starting a diagnostic trajectory for dementia: an integrative review. Aging Ment Health. 2023;27:862‐875. 35763442 10.1080/13607863.2022.2084505 PMC10166060

[11] Visser LNC , Fruijtier A , Kunneman M , et al. Motivations of patients and their care partners for visiting a memory clinic. A qualitative study. Patient Educ Couns. 2023;111:107693. 36913778 10.1016/j.pec.2023.107693

[12] van der Flier WM , Kunneman M , Bouwman FH , Petersen RC , Smets EMA . Diagnostic dilemmas in Alzheimer's disease: room for shared decision making. Alzheimers Dement. 2017;3:301‐304. 10.1016/j.trci.2017.03.008 PMC5651445 29067336

[13] Visser LNC , van Maurik IS , Bouwman FH , et al. Clinicians' communication with patients receiving a MCI diagnosis: the ABIDE project. PLoS One. 2020;15:e0227282. 31961882 10.1371/journal.pone.0227282 PMC6974141

[14] Abley C , Manthorpe J , Bond J , et al. Patients' and carers' views on communication and information provision when undergoing assessments in memory services. J Health Serv Res Policy. 2013;18:167‐173. 23864420 10.1177/1355819613479945

[15] Fruijtier AD , Visser LNC , van Maurik IS , et al. ABIDE Delphi study: topics to discuss in diagnostic consultations in memory clinics. Alzheimers Res Ther. 2019;11:77. 31472676 10.1186/s13195-019-0531-y PMC6717649

[16] Fruijtier AD , Visser LNC , Bouwman FH , et al. What patients want to know, and what we actually tell them: the ABIDE project. Alzheimers Dement. 2020;6:e12113. 10.1002/trc2.12113 PMC7744024 33344753

[17] Kunneman M , Pel‐Littel R , Bouwman FH , et al. Patients' and caregivers' views on conversations and shared decision making in diagnostic testing for Alzheimer's disease: the ABIDE project. Alzheimers Dement. 2017;3:314‐322. 10.1016/j.trci.2017.04.002 PMC5651429 29067338

[18] Visser LNC , Kunneman M , Murugesu L , et al. Clinician‐patient communication during the diagnostic workup: the ABIDE project. Alzheimers Dement. 2019;11:520‐528. 10.1016/j.dadm.2019.06.001 PMC6667786 31388556

[19] van der Schaar J , Visser LNC , Ket JCF , et al. Impact of sharing Alzheimer's disease biomarkers with individuals without dementia: A systematic review and meta‐analysis of empirical data. Alzheimers Dement. 2023;19:5793‐5794. 10.1002/alz.13410 37496313

[20] World Medical Association . World Medical Association Declaration of Helsinki: ethical principles for medical research involving human subjects. JAMA. 2013;310:2191‐2194. 24141714 10.1001/jama.2013.281053

[21] VERBI Software–Consult–Sozialforschung GmbH . MAXQDA 12, Software for Qualitative Data Analysis. VERBI Software–Consult–Sozialforschung GmbH; 2016.

[22] Hsieh HF , Shannon SE . Three approaches to qualitative content analysis. Qual Health Res. 2005;15:1277‐1288. 16204405 10.1177/1049732305276687

[23] Solomon A , Stephen R , Altomare D , et al. Multidomain interventions: state‐of‐the‐art and future directions for protocols to implement precision dementia risk reduction. A user manual for Brain Health Services—part 4 of 6. Alzheimers Res Ther. 2021;13:171. 34635167 10.1186/s13195-021-00875-8 PMC8507202

[24] Lohmeyer JL , Alpinar‐Sencan Z , Schicktanz S . Attitudes towards prediction and early diagnosis of late‐onset dementia: a comparison of tested persons and family caregivers. Aging Ment Health. 2021;25:832‐843. 32091238 10.1080/13607863.2020.1727851

[25] Yates J , Stanyon M , Samra R , Clare L . Challenges in disclosing and receiving a diagnosis of dementia: a systematic review of practice from the perspectives of people with dementia, carers, and healthcare professionals. Int Psychogeriatr. 2021;33:1161‐1192. 33726880 10.1017/S1041610221000119

[26] Joosten‐Weyn Banningh L , Vernooij‐Dassen M , Rikkert MO , Teunisse JP . Mild cognitive impairment: coping with an uncertain label. Int J Geriatr Psychiatry. 2008;23:148‐154. 17578843 10.1002/gps.1855

[27] Visser LNC , Pelt SAR , Kunneman M , et al. Communicating uncertainties when disclosing diagnostic test results for (Alzheimer's) dementia in the memory clinic: the ABIDE project. Health Expect. 2019;23:52‐62. 31638322 10.1111/hex.12964 PMC6978856

[28] Hendriksen HMA , van Gils AM , van Harten AC , et al. Communication about diagnosis, prognosis, and prevention in the memory clinic: perspectives of European memory clinic professionals. Alzheimers Res Ther. 2023;15:131. 37543608 10.1186/s13195-023-01276-9 PMC10404377

[29] Grad R , Légaré F , Bell NR , et al. Shared decision making in preventive health care: what it is; what it is not. Can Fam Physician. 2017;63:682‐684. 28904031 PMC5597010

[30] Entwistle VA , Cribb A , Watt IS . Shared decision‐making: enhancing the clinical relevance. J R Soc Med. 2012;105:416‐421. 23104944 10.1258/jrsm.2012.120039 PMC3480854

[31] Kunneman M , Smets EMA , Bouwman FH , et al. Clinicians' views on conversations and shared decision making in diagnostic testing for Alzheimer's disease: the ABIDE project. Alzheimers Dement. 2017;3:305‐313. 10.1016/j.trci.2017.03.009 PMC5651435 29067337

[32] Linden I , Wolfs C , Perry M , et al. Implementation of a diagnostic decision aid for people with memory complaints and their general practitioners: a protocol of a before and after pilot trial. BMJ Open. 2021;11:e049322. 10.1136/bmjopen-2021-049322 PMC8211080 34135053

[33] Berger ZD , Brito JP , Ospina NS , et al. Patient centred diagnosis: sharing diagnostic decisions with patients in clinical practice. BMJ. 2017;359:j4218. 29092826 10.1136/bmj.j4218

[34] Grill JD , Apostolova LG , Bullain S , et al. Communicating mild cognitive impairment diagnoses with and without amyloid imaging. Alzheimers Res Ther. 2017;9:35. 28472970 10.1186/s13195-017-0261-y PMC5418690

[35] Exalto LG , Hendriksen HM , Barkhof F , et al. van Leeuwenstijn‐Koopman M, et al. subjective cognitive decline and self‐reported sleep problems: the SCIENCe project. Alzheimers Dement. 2022;14:e12287. 10.1002/dad2.12287 PMC9107682 35603141

[36] van Gils AM , Visser LN , Hendriksen HM , Georges J , van der Flier WM , Rhodius‐Meester HF . Development and design of a diagnostic report to support communication in dementia: co‐creation with patients and care partners. Alzheimers Dement. 2022;14:e12333. 10.1002/dad2.12333 PMC9446898 36092691

[37] Pike KE , Cavuoto MG , Li L , Wright BJ , Kinsella GJ . Subjective cognitive decline: level of risk for future dementia and mild cognitive impairment, a meta‐analysis of longitudinal studies. Neuropsychol Rev. 2022;32:703‐735. 34748154 10.1007/s11065-021-09522-3

[38] van Maurik IS , Vos SJ , Bos I , et al. Biomarker‐based prognosis for people with mild cognitive impairment (ABIDE): a modelling study. Lancet Neurol. 2019;18:1034‐1044. 31526625 10.1016/S1474-4422(19)30283-2

[39] Kivipelto M , Mangialasche F , Ngandu T . Lifestyle interventions to prevent cognitive impairment, dementia and Alzheimer disease. Nat Rev Neurol. 2018;14:653‐666. 30291317 10.1038/s41582-018-0070-3

[40] Ngandu T , Lehtisalo J , Solomon A , et al. A 2 year multidomain intervention of diet, exercise, cognitive training, and vascular risk monitoring versus control to prevent cognitive decline in at‐risk elderly people (FINGER): a randomised controlled trial. Lancet. 2015;385:2255‐2263. 25771249 10.1016/S0140-6736(15)60461-5

[41] Tong A , Sainsbury P , Craig J . Consolidated criteria for reporting qualitative research (COREQ): a 32‐item checklist for interviews and focus groups. Int J Qual Health Care. 2007;19:349‐357. 17872937 10.1093/intqhc/mzm042

[42] Khalsa DS , Newberg AB . Spiritual fitness: a new dimension in Alzheimer's disease prevention. J Alzheimers Dis. 2021;80:505‐519. 33554917 10.3233/JAD-201433 PMC8075383

[43] Beuscher L , Beck C . A literature review of spirituality in coping with early‐stage Alzheimer's disease. J Clin Nurs. 2008;17:88‐97. 18298759 10.1111/j.1365-2702.2007.02126.x

[44] Altomare D , Molinuevo JL , Ritchie C , et al. Brain Health Services: organization, structure, and challenges for implementation. A user manual for Brain Health Services—part 1 of 6. Alzheimers Res Ther. 2021;13:168. 34635163 10.1186/s13195-021-00827-2 PMC8507194

[45] van Maurik IS , Visser LN , Pel‐Littel RE , et al. Development and usability of ADappt: web‐based tool to support clinicians, patients, and caregivers in the diagnosis of mild cognitive impairment and alzheimer disease. JMIR Form Res. 2019;3:e13417. 31287061 10.2196/13417 PMC6643768

