# Supplementary material for: The expectations and experiences of patients regarding the diagnostic workup at a specialized memory clinic: An interview study
Source: Health Expect. 2024 Mar 21;27(2):e14021. doi: 10.1111/hex.14021 (PMC10958124; doi:10.1111/hex.14021)
Supplement: Supplementary file 1 — Supporting information. [file HEX-27-e14021-s001.docx]

**Appendix A. Interview guide**

In this appendix, we present the global interview structure in English. Most interviews were conducted in Swedish, so nuances might be slightly different in the Swedish language. Follow-up questions were asked if needed, below we present only leading questions.

**INT 1.** First interview on the day(s) after the first visit to the clinic: the focus is on needs, wishes, preferences, expectations and prior knowledge.

Leading questions:

- How did you come to do this cognitive investigation?

- How did it feel to take the step to do this investigation?

- What is most important for you to get out of this investigation?

- How do you feel about this first part of the process, the visit(s) you have made to the clinic thus far?

- What do you think about the doctor's/staff’s communication?

- What would you have preferred in terms of communication?

- To what extent do you think that the investigation so far suits your wishes and needs?

- Can you tell us a little about what you already know about cognitive disease and dementia?

**INT 2.** Second interview within 2 weeks after diagnosis/test result disclosure consultation: the focus is on experiences during and (right) after receiving test results, and the (immediate) consequences/impact.

Leading questions:

- How did you experience your return visit to the clinic, to get the results of the investigation?

- How was the information given to you?

- How did you feel about the provided information?

- What do you think about the doctor's/staff’s communication?

- What would you have preferred in terms of communication?

- How has the investigation and the result affected you and your life?

- What negative effects, if any, can you think of?

- What positive effects, if any, can you think of?

- How has this process/result affected your view of yourself?

- How do you think about the future? Have the results of the investigation changed your thoughts about the future?

- To what degree do you think your wishes and needs have been met?

- What could the clinic have done different to better meet your wishes and needs?

**INT 3.** Third interview around three months after the diagnostic trajectory and receiving results: focus on long-term impact/consequences and evaluation of memory clinic care.

Leading questions:

- How do you feel now when you look back on the investigation and the results you received?

- What do you think about the doctor's/staff’s communication?

- What could the clinic (staff) have been done different to better meet your wishes and needs?

- How has the result of the cognitive investigation and the visits to the clinic affected you and your

life?

- What negative effects, if any, can you think of?

- What positive effects, if any, can you think of?

- How are your interactions with others now compared to before?

- Have you told anyone about your diagnosis/problems? Do you feel that you have had enough?

much information and knowledge to be able to discuss your diagnosis/results with others?

Questions about spirituality:

- What does spirituality mean to you?

- How important is spirituality to you?

- How does spirituality help you deal with events or difficulties in life, such as cognitive impairment?
